# Bacterial Genotoxin-Induced DNA Damage and Modulation of the Host Immune Microenvironment

**DOI:** 10.3390/toxins12020063

**Published:** 2020-01-21

**Authors:** Océane C.B. Martin, Teresa Frisan

**Affiliations:** 1 Univ. Bordeaux, INSERM, UMR1053 Bordeaux Research in Translational Oncology, BaRITOn, 33320 Bordeaux, France; oceane.martin@u-bordeaux.fr; 2 Department of Cell and Molecular Biology Karolinska Institutet, 17177 Stockholm, Sweden; 3 Umeå Center for Microbial Research (UCMR), Umeå University, 90187 Umeå, Sweden; 4 Department of Molecular Biology, Umeå University, 90187 Umeå, Sweden

**Keywords:** bacterial genotoxins, DNA damage, DNA damage response, immune response, immunomodulation, senescence

## Abstract

Bacterial genotoxins (BTGX) induce DNA damage, which results in senescence or apoptosis of the target cells if not properly repaired. Three BTGXs have been identified: the cytolethal distending toxin (CDT) family produced by several Gram-negative bacteria, the typhoid toxin produced by several *Salmonella enterica* serovars, and colibactin, a peptide-polyketide, produced mainly by the phylogenetic group B2 *Escherichia coli*. The cellular responses induced by BTGXs resemble those of well-characterized carcinogenic agents, and several lines of evidence indicate that bacteria carrying genotoxin genes can contribute to tumor development under specific circumstances. Given their unusual mode of action, it is still enigmatic why these effectors have been acquired by microbes and what is their role in the context of the biology of the producing bacterium, since it is unlikely that their primary purpose is to induce/promote cancer in the mammalian host. In this review, we will discuss the possibility that the DNA damage induced by BTGX modulates the host immune response, acting as immunomodulator, leading to the establishment of a suitable niche for the producing bacterium. We will further highlight open questions that remain to be solved regarding the biology of this unusual family of bacterial toxins.

## 1. Bacterial Genotoxins

Bacteria have acquired a broad array of toxins, which are key virulence factors to ensure host colonization, invasion, and escape from the immune response, allowing replication and spread to a new host or the surrounding environment. Toxins target all the key compartments of the host eukaryotic cells: plasma membrane, cytoskeleton, protein synthesis, intracellular signaling, and even DNA [1]. The latter activity is mediated by a family of toxins that are functional homologous to mammalian DNAse I: the cytolethal distending toxin (CDT) and the typhoid toxin, which promote DNA single (SSB) and double strand break (DSB) in the target cells [2]. These toxins are produced by several Gram-negative bacteria, including *Escherichia coli, Aggregatibacter actinomycetemcomitans*, *Shigella dysenteriae*, *Haemophilus ducreyi*, *Helicobacter sp*., *Campylobacter sp*., typhoidal, and some non-typhoidal *Salmonella* serovars. In this review, we have used the CDT nomenclature proposed by Thelestam et al. and Jinadasa et al. where each CDT is specified by defining the producing bacterium using the first capitalized letter of the genus followed by the first three letters of the species name in lower case before CDT and, if necessary, the strain number or other common designation after CDT (e.g., EcolCDT-I for the CDT I variant produced by *E. coli*, or HducCDT for the toxin produced by *H. ducreyi*) [3,4].

An additional effector that causes DNA damage, by inducing DNA interstrand cross-links is colibactin, the product of the *psk* island, present in group B2 *E. coli, Klebsiella pneumoniae*, *Enterobacter aerogenes*, and *Citrobacter koseri* [5].

Collectively these toxins will be defined in this review as bacterial genotoxins (BTGX).

The puzzling question that still needs to be answered regarding these very unusual effectors, which act as *bona fide* carcinogens [2], is their role in the context of the biology of the producing bacterium. This review will try to propose an answer to this question. An answer that may be extended to other bacteria that induce DNA damage: (i) directly via activation of host endonucleases XPF and XPG (*Helicobacter pylori*); (ii) indirectly by downregulation of components of pathways that repair oxidative stress-induced damage or nucleotide mismatch (e.g., *H. pylori*), or inhibition of effectors of the DNA damage response (DDR) (e.g., *Chlamydia trachomatis, Listeria monocytogenes*) [6].

### 1.1. Cytolethal Distending Toxins And Typhoid Toxin Structure

**Figure 1:**
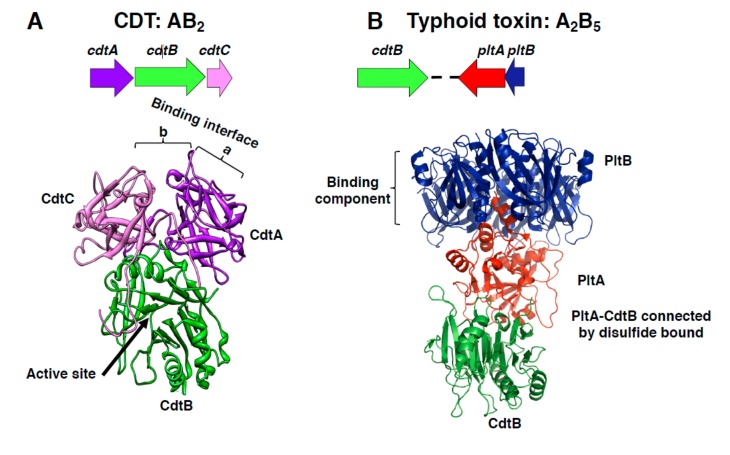
Cytolethal distending toxin (CDT) and typhoid toxin structure. (**A**) Schematic representation of the CDT operon from *H. ducreyi* and the crystal structure of the holotoxin, adapted from Nesic et al. [7], PDB access number: 1SR4. The CdtB is the active subunit, while the CdtA and CdtC accessory subunits contribute to the binding interface, composed by the aromatic patch in CdtA (a) and an adjacent deep groove (b). (**B**) Schematic representation of the *Salmonella enterica* serovar Typhi islet encoding for the typhoid toxin genes and crystal structure of the holotoxin, adapted from Song et al., PDB access number: 4K6L [10]. This toxin contains two active subunits: CdtB, homologous to mammalian DNase I, a characteristic shared with CDTs, and the ADP ribosyl transferase PltA, and they are linked to each other by a disulfide bound. The binding moiety is formed by a pentameric disc made by five PltB monomers.

The members of the CDT family are AB_2_ trimers [7], composed of the active (A) subunit CdtB, and two binding moieties (B), CdtA and CdtC, encoded by a single operon (Figure 1A). The sequence homology between the *cdtA*, *cdtB,* and *cdtC* genes among different bacteria species is variable, and several CDTs have been identified even within the same species (e.g., *E. coli*). The most conserved gene is the *cdtB*, encoding for the active subunit (45% sequence identity), while the genes encoding for the CdtA and CdtC subunits are the most divergent [8]. CdtA and CdtC subunits are lectin-type molecules. The crystal structure of the HducCDT shows that they present structural homology with the B-chain repeats of the plant toxin ricin, and form an interface, constituted by the large aromatic patch in CdtA and an adjacent deep groove on the protein surface, required for interaction with the host cells [7,9] (Figure 1A).

The CdtB subunit adopts the canonical four-layered fold of the DNase I family: a central 12-stranded β-sandwich packed between outer α-helices and loops on each side of the sandwich. The two catalytic histidine residues are conserved between HducCdtB (His160 and His274) and DNAse I (His134 and His252) as well as the three residues responsible for binding to DNA (Arg 144, Asn 201, and Arg 117 in CdtB, corresponding to Arg 111, Asn 170, and Arg 41 of the DNAse I) [7].

The typhoid toxin genes, present in *S. enterica* serovar Typhi, Paratyphi, Schwarzengrund, 9, 12:l, v:-, Bredeney, and *S. enterica* subspecies *arizonae*, *diarizonae*, and *javiana*, encode for an A_2_B_5_ protein complex (Figure 1B) [10,11,12,13], where CdtB and PltA subunits carry the enzymatic activity (A), and the pentameric ring formed by the PltB subunit represents the binding unit (B) [10]. Interestingly, the three typhoid toxin genes are transcribed from two different promoters (one for the operon encoding for PltB and PltA, and one encoding for the CdtB subunit) within a pathogenicity islet in *S*. Typhi [14] (Figure 1B). Crystallographic studies demonstrate that the PltA subunit is structurally similar to the pertussis toxin S1, an ADP ribosyl transferase, while CdtB is similar to the CdtB present in the CDT holotoxins [10]. The two active subunits of the typhoid toxin have a minimal interacting interface and are linked by a disulfide bond between Cys 214 of PltA and Cys 269 of CdtB [10].

Recently, a distinct form of typhoid toxin where the CdtB and PltA subunits are associated with a district pentameric ring formed by the PltC subunit has been described in *S.* Typhi [15].

In spite of the relative low aminoacid identity of the active subunit and the different holotoxin composition of CDTs and the typhoid toxin, comparison of crystal structures demonstrated that the HducCdtB, AactCdtB, EcolCdtB-II, and the typhoid toxin CdtB have a very conserved structure, and all target the eukaryotic cellular DNA. Conversely, the cellular targets of PltA have not been yet identified [10]. For further functional and structural comparison between the different members of this toxin family, we refer the reader to a very comprehensive review by Pons et al. [16].

Among all bacterial AB toxins, CDTs and the typhoid toxin are the only effectors that require translocation into the nuclear compartment to interact with their target: the cellular DNA. In this review we will focus on the DNA damaging effects of these toxins and their modulation of the host microenvironment, specifically on host immune responses, and we refer to other comprehensive reviews for a detailed analysis of the internalization pathways [2,17,18,19].

### 1.2. CDTs Activity

Most experimental data concerning the type and kinetics of the DNA damage induced by these toxins have been generated using CDT as the model toxin.

Fedor and colleagues have performed a detailed analysis of the kinetics and type of DNA damage induced by the EcolCDT-I and showed that low toxin doses (50 pg/mL) induce SSBs 3 h–6 h post-intoxication, which are converted into DSBs during the S phase of the cell cycle due to inhibition of the replicative fork as a consequence of unrepaired SSBs [20]. Direct induction of DNA DSBs was instead observed in cells exposed to high toxin doses (above 75 ng/mL) independently of the cell cycle phase, possibly due to the induction of juxtaposing SSBs on opposite strands. These observations reconcile some discrepancies in the literature, where transit through the S phase of the cell cycle was demonstrated to be required for the activity of EcolCDT [21,22], while other authors demonstrated that the HducCDT toxin also induces DNA damage in non-proliferating cells [23].

It is noteworthy that the enzymatic activity of the CdtB subunit on purified DNA plasmid was estimated to be 10,000 times lower than the purified human or bovine DNase I [24]. Similarly, microinjection of 40 μg/mL of the HducCDT was required to promotes chromatin condensation and nuclear blebbing in HeLa cells, while a similar effect was obtained by microinjection of 40 pg/mL of purified bovine DNAse I [23]. Based on this low efficiency and on the observation that DNase I belongs to a broad superfamily, which includes several Mg^2+^-dependent phosphoesterases such as the inositol polyphosphate 5-phosphatase, it was suggested that CdtB might have an additional enzymatic activity [25]. In line with this hypothesis, Shenker and colleagues reported that the CdtB subunit from *A. actinomycetemcomitans* exhibits a PI-3,4,5-triphosphate (PI-3,4,5-P_3_) phosphatase activity in vitro [26]. Correspondingly, AactCDT intoxication significantly reduces the intracellular levels of PI-3,4,5-P_3_ in several human T cell lines expressing high intracellular levels of PI-3,4,5-P_3_, due to mutations in the *PTEN* and/or *SHIP1* genes, but has no effect on the HUT78 cells, which contain low levels of PI-3,4,5-P_3_ [26]. Thus, CdtB can exert dual activities: DNAase and inositol polyphosphate 5-phosphatase. The latter can be exerted under specific circumstances (e.g., high intracellular levels of PI-3,4,5-P_3_), while the genotoxic activity is context-independent, since it is systematically observed in all the cell lines tested, with the only limitation being the species specificity barrier [4,27].

The genotoxic activity of CDT was shown to promote acquisition of carcinogenic traits (genomic instability, enhanced mutation frequency, growth in anchorage independent manner) in vitro in cells chronically exposed to sublethal doses of a functional CDT [28,29]. This was confirmed in several in vivo models where long-term colonization with CDT-producing *C. jejuni* or *H. hepaticus* induces gastric dysplasia in NFκB deficient mice or hepatic dysplasia in A/JCr mice, respectively [30,31], while infection with wild type *C. jejuni*, but not the *cdtB* mutated isogenic strain, promotes cancer in the colitis-associated colorectal cancer *Apc^Min/+^*/DSS (Dextran Sulfate Sodium) mouse model [32]. The CDT carcinogenic potential is supported by epidemiological data, demonstrating a higher frequency of CDT-producing *E. coli* in the mucosa of colorectal cancer (CRC) and Inflammatory Bowel Disease (IBD) patients compared to control subjects [33]. In addition, *S.* Typhi is the only genotoxin-producing bacterium associated with an increased risk of developing hepatobiliary carcinoma in humans [34].

### 1.3. Colibactin

Colibactin was first identified in 2006 as the product of a 54 Kb genomic island designated as *pks* in the *E. coli* meningitidis strain IHE3034 [35]. *pks* positive *E. coli* are frequent colonizers of the newborn gut in human, enriched in the microbiota of IBD and CRC patients, and this island is also present in the probiotic strain Nissle 1917 [33,36,37,38,39,40].

The *pks* island harbors 19 genes (named *clbA* to *clbS*), which encode for three nonribosomal peptide megasynthetases (NRPS, CLBH, ClbJ, and ClbN), three polyketide megasynthetases (PKS, ClbC, ClbI, and ClbO), two hybrid NRPS/PKS megasynthetases (ClbN and ClbK), and nine accessory, tailoring and editing enzymes [35].The products of this genomic island are hybrid polyketide-non-ribosomal peptides and have been shown to exert different functions: from induction of DNA damage by inducing DNA interstrand cross-links to antimicrobial (indirectly via production of the microcins MccH47 and MccM) and analgesic (via production of the lipopeptide C12AsnGABA*OH*) activity [35,41,42,43,44], highlighting the complexity of this region [5].

Focusing on the DNA damaging activity, *pks* positive *E. coli* have been shown to induce DNA damage in vitro and in vivo [35,45]. This genotoxic activity is dependent on live bacteria and requires a direct contact with the host cells. Furthermore, bacterial supernatant cannot induce the toxin effect, suggesting that colibactin is directly injected into the host cells [35].

The genotoxic activity of *pks* positive *E. coli* promotes acquisition of carcinogenic traits (genomic instability, enhanced mutation frequency, anchorage independent growth) in vitro [45], enhances tumor formation in germ free IL10 deficient mice treated with the pre-carcinogen azoxymethane (AOM) [37], impairs the intestinal homeostasis and consequently alters oral tolerance in rats upon neonatal colonization [46,47].

A common cellular response to all BTGXs is the activation of the DNA damage response (DDR), which is a highly conserved danger sensing pathway with the main function to preserve genome integrity. In the next section, we will briefly summarize the key steps and outcomes of the cellular responses to the types of DNA damage induced by BTGXs: DNA SSBs, DSBs, and DNA interstrand cross-links, focusing on the effectors that are relevant in the context of BTGXs biology. The detailed characterization of these pathways has been excellently reviewed elsewhere [48,49,50,51,52].

## 2. The DNA Damage Response

**Figure 2:**
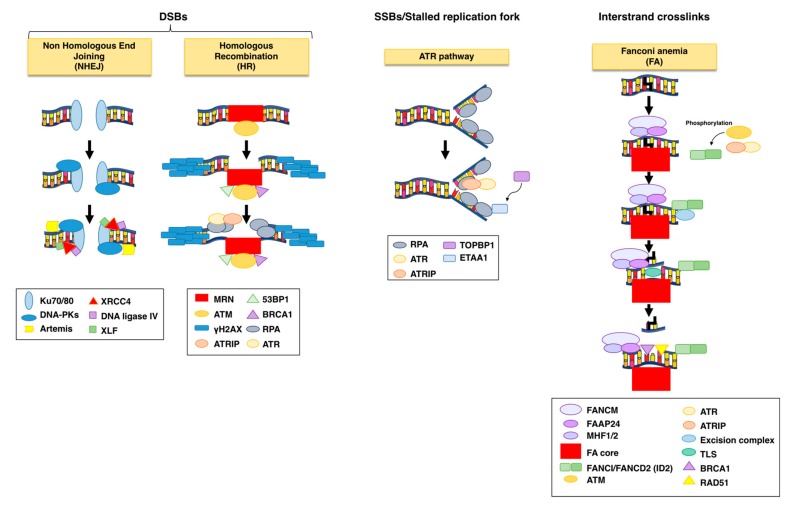
DNA repair mechanisms activated upon bacterial genotoxin (BTGX)-induced DNA damage. DNA double-strand breaks (DSBs) are repaired via non-homologous end joining (NHEJ) or homologous recombination (HR), while single-strand breaks (SSB) associated with stalled replication fork are repaired through the Ataxia Telangectasia Mutated (ATM)and the ATM-Rad3 related (ATR) pathway. Interstrand cross-links are sensed and repaired via the Fanconi anemia pathway. The NHEJ pathway starts with the recognition of the break by the Ku heterodimer, which will lead to the recruitment of DNA-dependent protein kinase (DNA-PK), followed by re-localization of ARTEMIS at the damaged site. This protein promotes a limited end processing required for the ligation of the broken DNA end by the XRCC4/Lig4 ligase, aided by the stimulatory factor XLF. In the presence of a DNA template such as sister chromatid, the preferred repair system is the error-free HR. Key for the HR pathway is the recruitment of the MRE11-RAD50-NBS1 (MRN) complex on the damaged site, which will help the localization and activation of ATM. The optimization of the ATM-MRN interaction is assisted by 53BP1 and BRCA1 and will result on the phosphorylation of the histone H2AX (γH2AX). The damage DNA will be resected to expose single strand DNA, which will be stabilized by the replication protein A (RPA) complex and recruitment of ATR-interacting protein (ATRIP)/ATR. Strand invasion of the sister chromatid, assisted by the BRCA2-dependent recruitment of RAD51, will finalize the HR process. The SSBs and exposure of ssDNA at the stalled replication fork will be recognized by the replication protein A (RPA) complex, which forms a platform for the recruitment ATRIP/ATR and other proteins, such as TOPBP1 and ETAA1 required for proper ATR activation. Interstrand cross-links are sensed by the FANCM-FAAP24-MH1/MH2 complex, which recruits the Fanconi anemia (FA) core. This complex, assisted by ATM and ATR, promotes phosphorylation and mono-ubiquitination of the FANI-FAND2 (ID2) heterodimer. ID2 is key for the repair mechanism, since it will recruit: (i) the excise on complex to cut the regions flanking the damaged site; (ii) translesion (TLS) polymerases to fill in the gap on the non-cleaved site; (iii) homologous recombination factors, such as RAD51, BRCA1 to finalize the DNA repair.

**Figure 3:**
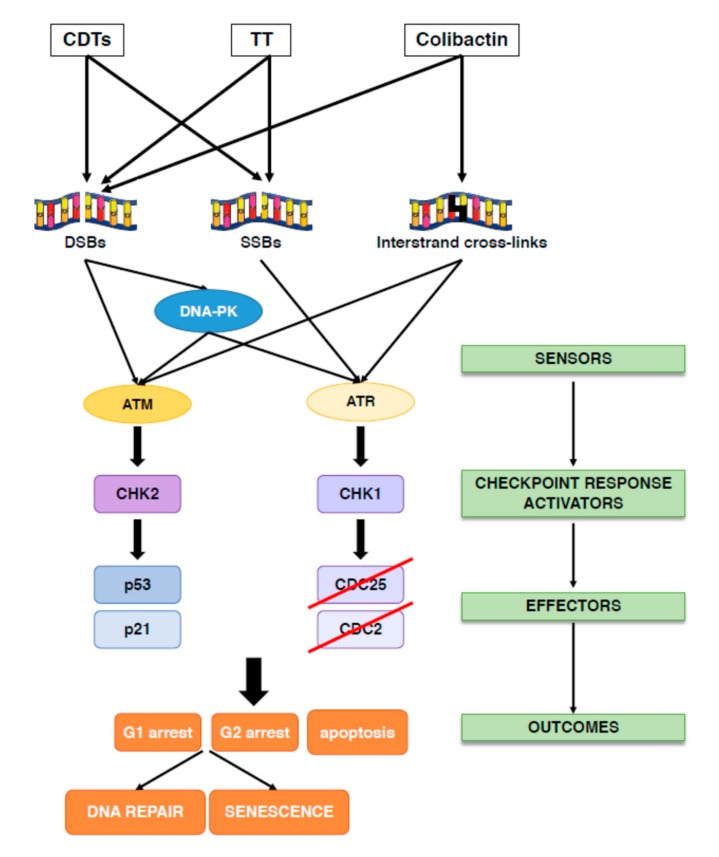
DNA damage response (DDR) activation in response to bacterial genotoxins. Cytolethal distending toxin (CDT) and typhoid toxin (TT) are AB toxins able to induce SSBs at low dose and DSBs at high dose, while colibactin is a polyketide toxin that induces interstrand cross-links, which can be further converted into DSBs. Once the damages are detected by specific sensors (DNA-PK, ATM and ATR), the checkpoint responses are activated leading to cell cycle arrest to allow DNA repair. DNA-PK and ATM are sensors for DSBs, while ATR senses SSBs. Both ATM and ATR are activated in response to interstrand cross-links. ATM and DNA-PK activation phosphorylates the kinase CHK2 leading to the phosphorylation and stabilization of the tumor suppressor p53 and increased expression of its downstream effector p21, blocking cell proliferation in the G1 phase of the cell cycle. ATR and DNA-PK phosphorylate the kinase CHK1, which blocks cell proliferation in the G2 phase of the cell cycle by inactivating the CDC25 phosphatase, resulting in hyperphosphorylation and inactivation of the cyclin dependent kinase CDC2. Failure to repair DNA will promote either senescence or apoptosis in a cell type dependent manner.

The overall principle of the cellular response to DNA damage includes: (i) sensing the damage; (ii) blocking cell division (activation of checkpoint responses); (iii) activating DNA repair mechanisms, as summarized in Figure 2; Figure 3.

Three related kinases, the DNA-dependent protein kinase (DNA-PK), the Ataxia Telangectasia Mutated (ATM), and the ATM-and Rad3 related (ATR) sense the damaged DNA and coordinate cell cycle arrest and repair [49]. Specifically, DNA-PK and ATM are key regulators of the DDR in response to DSBs, while ATR responds to various types of DNA lesions associated with alteration of the DNA structure or stalling of the replication fork (Figure 2).

DNA-PK has as major role in promoting Non Homologous End Joining (NHEJ) repair by ligating the two broken DNA ends without requiring a repair template. NHEJ is the major repair pathway outside the S and G2 cell cycle phases.

During NHEJ, the DSB breaks are recognized by the Ku heterodimer (Ku70 and Ku80), which is loaded within seconds on the damaged site, leading to the recruitment of two molecules of DNA-PK. This interaction stimulates the kinase activity of DNA-PK, promoting phosphorylation in trans across the DSB [53]. The function of this complex is to tether the two DNA ends together and form a scaffold for the recruitment and activation of the downstream repair effectors, which can process the DNA termini to remove lesions that cannot be ligated (e.g., Artemis, a 5’ to 3´exonuclase), add nucleotides to fill eventual gaps (DNA polymerases μ and λ), and finally ligate the two ends (DNA ligase IV and XRCC4 in complex with XRCC4-like factor XLF) [53,54], as summarized in Figure 2. Since NHEJ acts mainly in absence of a template it is considered error-prone, differently from homologous recombination (HR), which instead ensures a faithful repair.

ATM is the master regulator for DNA DBS repair via HR. This pathway starts with a rapid poly(ADP-ribosyl)ation of histones around the DNA lesion to form linear and branched polymers, known as poly(ADP-ribose) (PAR), mainly induced by the poly(ADP-ribose) polymerases (PARP) 1, 2, and 3. Due to the high negative charges, PAR relaxes chromatin by electron repel and functions as docking sites for the recruitment of chromatin remodelers and the MRE11-RAD50-NBS1 (MRN) complex. This complex tethers the DNA ends and is essential for the localization and activation of ATM at the damaged site [55]. Optimization of the ATM-MRN interaction and its retention at the damage site is assisted by 53BP1 and BRCA1, resulting in activation of the checkpoint responses via phosphorylation of the downstream effectors CHK2 and p53. The outcome of this response is induction of cell cycle arrest in different phases of the cell cycle (G1, S, and G2), and repair via the HR pathway [56,57].

One of the first downstream targets of ATM is the histone variant H2AX, phosphorylated on S139 (defined as γH2AX), which extends for 1-2 megabases around the lesion, forming discernible nuclear foci easily detectable by fluorescent microscopy, and therefore commonly used as surrogate marker for DNA DSB [48,58] (Figure 2). γH2AX and the MRN complex form a platform for the sequential recruitment and retention of repair factors that promote: (i) resection of the DSB to produce single stranded DNA (ssDNA) by CtlP, aided by BRCA1; (ii) protection and stabilization of the ssDNA by the RPA complex (RPA1, RPA2, and RPA3); (iii) strand invasion into the homologue sequence of the sister chromatid by the recombinase RAD51, aided by BRCA2; (iv) extension of the 3′ invading strand by DNA polymerase δ, formation of the D loop and, finally, resolution of the break [48,56].

ATR responds to various types of DNA lesions by interacting with ssDNA. These lesions can be due to nucleotide modification by genotoxic agents (e.g., thymidine dimers caused by UV radiation) or stalling the replication fork progression [48,59]. ssDNA is recognized by the replication protein A (RPA) complex, which prevents the generation of DNA secondary structures and protects ssDNA from degradation. The RPA complex forms a platform for the recruitment of ATR-interacting protein (ATRIP), bound to ATR, and additional factors necessary for the ATR kinase activation (Figure 2). [60]

Independently on the ATR binding, the presence of ssDNA bound-RPA recruits the RAD17-RCF2-5 clamp loader, followed by the proliferating cell nuclear antigen (PCNA)-related 9-1-1 clamp, consisting of Rad9, Rad1, and Hus1 subunits, and recruitment of TOPBP1, which is required for full activation of the ATR kinase activity. A second ATR activator, ETAA1 is recruited to the DNA lesion by direct binding to RPA [48,49,61,62,63]. Once primed, recruited, and stimulated, ATR phosphorylates a series of downstream effectors, which results in activation of checkpoint responses (CHK1) and DNA repair (e.g., FANCI and Polη).

The Fanconi anemia (FA) pathway is crucial for repairing DNA interstrand cross-links (ICL), and it is activated and coordinated by a family of approx. 22 genes (FANCA /B /C /D1 /D2 /E /F /G /I /J /L /M /N /O /P /Q /R /S /T /U /V and /W). Germline mutation in any of these genes causes Fanconi anemia, a rare genetic disease [50].

The damaged site is recognized by the FANCM-FAAP24-MH1/MH2 complex, which recruits the FA core (FANCA, FANCB, FANCC, FANCE, FANCF, FANCG, FANCL, FANCT, and the associated effectors FAAP20 and FAAP100) [50,51]. This complex structure, in association with ATM and ATR/ATRIP, promotes phosphorylation and mono-ubiquitination (via the Ubiquitin E3 ligase FANCL) of FAND2 and FANCI proteins, which dimerize to form the ID2 heterodimer. ID2 is central for the recruitment of the DNA repair effectors, including: (i) endonucleases (FAN1, XPF/ERCC1, MUS81/EME1, bound to the scaffold protein SLX4) to perform two incisions flanking the crosslinked site on the same strand and unhook the ICL [64]; (ii) translesion (TLS) polymerases (REV1 and Polζ ) to fill in the gap on the non-cleaved site; (iii) homologous recombination factors (RAD51, BRCA1, BRCA2, PALB2) to repair the DNA break [48,50,51,52], as summarized in Figure 2.

Cells that are not able to complete a proper DNA repair will undergo either apoptosis or senescence (Figure 3). Both outcomes prevent cells from acquiring genomic instability, one of the enabling characteristics of cancer, and are therefore considered a tumorigenesis barrier [65,66,67].

Senescence is characterized by a permanent cell cycle arrest, induced by activation of several cyclin-dependent kinase inhibitors, mainly CDKN2A (p16^INK4a^ or p16), CDKN2B (p15^INK4b^, p15), and CDKN1A (p21^CIP^, p21), and an active metabolic state resulting in secretion of a broad panel of mediators (senescence-associated secretory phenotype or SASP) [68,69,70,71]. Most of the secreted factors possess a pro-inflammatory profile, including cytokines (IL1α, IL6, IL8), growth factors (HGF, GM-CSF), and metalloproteases (MMP1-1 and MMP-3). SASP is mainly regulated by the transcription factors NFκB [72], triggered by the ATM-dependent DDR, as well as by C/ERBPβ and MAPK p38 [72].

The tumor suppressor p53 is one of the key proteins in determining cell fate by activating a complex gene network resulting in qualitative different outputs (apoptosis versus cell cycle arrest and/or senescence) depending on the cell type and the stimulus. The different outcomes may be dependent on a different pattern of p53 post-translational modifications that can alter p53 affinity for its target genes. Indeed, phosphorylation on Ser46 and acetylation on Lys120 stimulates apoptosis, while acetylation of Lys320 promotes activation of cell cycle arrest [73,74]. Both outcomes strongly effect the remodeling of the host microenvironment.

Exposure to CDT, typhoid toxin and colibactin or their producing bacteria activate the classical DDR responses in vitro and in vivo (Figure 3).

Thus, the response to CDT is ATM dependent, since a delayed effect was observed in ATM deficient cell lines [75]. Early CDT intoxication or infection with typhoid toxin-producing *Salmonella* is associated with: (i) DNA fragmentation; (ii) phosphorylation and γH2AX foci formation; (iii) re-localization of the DNA repair proteins, such as MRE11, NBS1, RAD50, 53BP1, and RPA at the sites of the damaged DNA; (iv) ATR-dependent replicative stress signaling; (v) dependency on functional BRCA2 and RAD51, (vi) activation of NHEJ, HR, and Fanconi-dependent repair pathways; (vii) activation of the tumor suppressor p53 and its transcriptional target, the cyclin-dependent kinase inhibitor p21; (viii) phosphorylation of the checkpoint kinases CHK1 and CHK2; (ix) inactivation of CDC25 phosphatases and accumulation of the inactive hyper-phosphorylated form of CDC2; (x) induction of senescence or apoptosis [14,20,22,23,75,76,77,78,79,80,81,82,83,84,85,86,87]

In the context of exposure to CDT, the choice between cell death and cell cycle arrest, and eventually acquisition of a senescence phenotype, has been shown to be dependent also on the ATM-dependent activation of the small GTPase RHOA, and downstream activation of the MAPK p38 [88,89]. Interestingly, RHOA activation has not been detected in intoxicated lymphoblastoid B cell lines, which are of hematopoietic origin [88] and undergo apoptosis upon intoxication [75]. In addition, an inside-out activation of integrin β1 has been shown to prolong survival of the intoxicated cells in anchorage independent conditions [90].

The detection of the DNA damage and activation of the DDR in vitro is paralleled by the detection of DNA fragmentation, γH2AX and senescence in mice infected with a *Salmonella* strain carrying a functional typhoid toxin (Martin et al. submitted).

Infection of cells with *pks* positive *E. coli* activates the classical DNA interstrand cross-links repair pathway resulting in: (i) monoubiquitination and recruitment of FANCD2 at stalled replication forks; (ii) ATR-dependent phosphorylation of CHK1 and RPA; (iii) activation of the DSB repair pathway, including phosphorylation of ATM, H2AX, and CHK2, and 53BP1 foci formation in response to replication stress-mediated DSBs; (iv) induction of cell cycle arrest and senescence [35,41].

The activation of the DDR response (assessed by detection of γH2AX) and genomic instability (assessed by detection of anaphase bridges) was also detected in vivo upon administration or neonatal colonization with *pks* positive *E. coli* [45,46].

## 3. Modulation of Immune Response by the BTGXs-induced DNA Damage

### 3.1. The Fine-Tuning of the Immune Response

The immune response (IR) is another complex system devoted to detection and elimination of threats and essential for the maintenance of the organism homeostasis. In this section, we will briefly outline the types of responses that are activated to eliminate threats. We will focus on the key cellular and molecular effectors that will be relevant to discuss the cross-talk between DDR and IR in the context of BTGXs.

Similar to activation of the DDR in response to DNA damage, perturbation of the organism homeostasis, either caused by infection or tissue stress or damage, is: (i) sensed (e.g., through pathogen or danger recognition receptors); (ii) transduced to relay the degree and type of insult (e.g., cytokine production); (iii) eliminated via activation of an array of effector cells and molecules. Finally, the immune response has to be terminated, concomitantly with the repair of the collateral damage [91].

**Figure 4:**
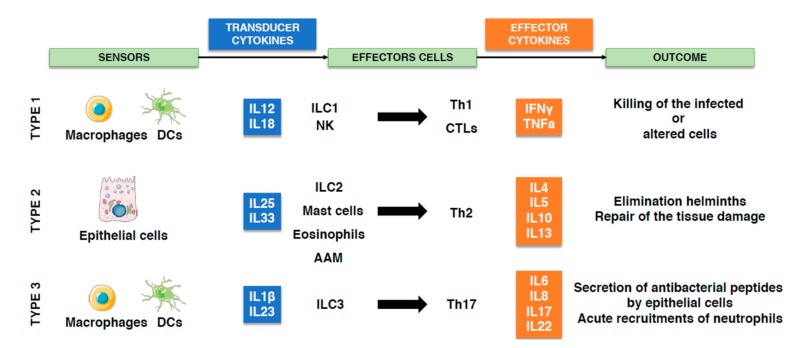
The three different types of immune response. Activation of the immune response starts with detection of a danger by specific receptors, leading to secretion of transducer cytokines. These mediators activate the effectors cytokines and cells that will eliminate the threat. In response to altered cells (e.g., tumor) or infection with intracellular microorganisms, the type 1 immune response will be coordinated by the release of IL12 and IL18 by macrophages and dendritic cells (DCs), leading to innate lymphoid cells (ILC)1 and natural killer (NK) cells activation, followed by activation and recruitment of CD4 Th1 lymphocytes and CTLs. In response to helminths, epithelial cells secrete IL25 and IL33 and initiate the type 2 immune response, leading to the activation of ILC2, mast cells, eosinophils, and consequently Th2 lymphocytes and alternatively activated macrophages (AAM). Upon infection with extracellular microorganisms, macrophages and DCs secrete IL1β and IL23, initiating the type 3 immune response that activate ILC3, Th17 lymphocytes, leading to acute neutrophils influx at the site of infection.

Based on threat and the type of effectors that are activated, three canonical types of response have been identified: type 1 against intracellular threats (viruses, intracellular bacteria, and cancer cells); type 2 against large extracellular threats (helminths); type 3 against extracellular microorganisms (extracellular bacteria and fungi) [92,93] (summarized in Figure 4). In all cases, successful elimination of the threat depends on a coordinated activity between innate and adaptive immune cells [94].

The output of the type 1 response is to promote an alert immunological state through secretion of IL12 and IL18 from dendritic cells (DCs) and macrophages. This results in a cascade of events leading to enhanced DCs antigen presenting capacity, activation of innate lymphoid cells (ILC)1 and natural killer (NK) cells, followed by activation of CD4 T helper (Th)1 and cytotoxic T (CTLs) lymphocytes. The final outcome is the killing of infected or altered cell(s). Key effector cytokines of the type 1 response are IFNγ and TNFα [92,95,96].

The type 2 response develops in response to large organisms (e.g., helminths) and activate tissue repair mechanisms to contain and eliminate them. This response is initiated by production of IL25 and IL33 by epithelial cells, leading to activation of ILC2, mast cells, and eosinophils, followed by activation of Th2 and alternatively activated macrophages (AAM). Key effectors cytokines are IL4, IL5, IL10, and IL13 [92,95,97,98].

The type 3 response is initiated by IL1β and IL23 secretion from DCs and macrophages leading to activation of ILC3 and Th17 and secretion of antibacterial peptides by epithelial cells. The key effector cytokines of this response are IL6, IL8, IL17, and IL22, resulting in an acute neutrophil recruitment [92,95,99].

Interestingly, the type of response activated upon an insult may not be dictated only by the nature of insult itself (e.g., intracellular versus extracellular microorganism), but also by the tissue affected. This integration can activate the best mechanisms to eliminate the threat, and simultaneously avoid unnecessary destruction that may compromise the tissue/organ function [100]. The tissue/context specificity may also apply to the outcome induced by infection with BTGXs producing bacteria, as discussed in the next section.

### 3.2. BTGXs-Induced DNA Damage and the Immune Response

Considering that DDR and IR are two conserved systems aimed at maintaining the organism homeostasis, it is possible that the cross-talk between these two responses is key in understanding why certain bacteria have acquired and possibly horizontally transferred [101,102,103] unusual effectors that cause DNA damage in eukaryotic cells, such as BTGXs.

**Figure 5:**
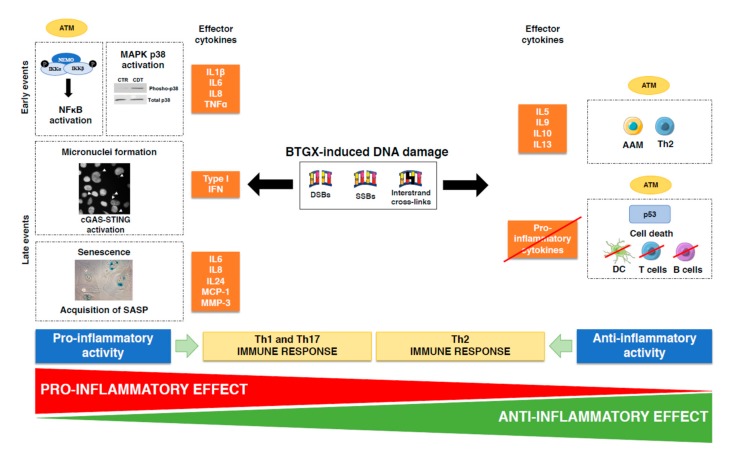
Cross-talk between activation of the DDR and the IR upon infection with genotoxin-producing bacteria. Infection with genotoxin-producing bacteria induce DDR activation. However, presence of damaged DNA will also be perceived as dangerous and trigger the host immune response, resulting in either pro-inflammatory or anti-inflammatory outcomes. Three events are linked to the pro-inflammatory profile of bacterial genotoxins (BGTX): (i) ATM-dependent NFκB activation and parallel MAPK p38 phosphorylation and consequent production of pro-inflammatory cytokines: IL1β, IL6, IL8, and TNFα; (ii) formation of micronuclei, which activates the cGAS-STING pathway, leading to secretion of type I IFN; (iii) acquisition of a senescence-associated inflammatory phenotype (SASP) leading to secretion of IL6, IL8, IL24, MCP-1, and MMP3. These pro-inflammatory cytokines will mainly coordinate activation of Th1 and Th17 responses. The anti-inflammatory properties of BTGXs are associated with: (i) ATM-dependent activation of T regulatory cells, alternatively activated macrophages (AAM) and Th2 cytokines (IL5, IL9, IL10, and IL13); (ii) induction of cell death and inhibition DCs, macrophages, and T lymphocytes activation.

This section will analyze the cross-talk between the DDR and the modulation of the host response in the context of intoxication or infection with BTGX producing bacteria in vitro and *in vivo*. The key results are summarized in Figure 5. We will not discuss the role of bacterial genotoxins in carcinogenesis, which has been exhaustively reviewed elsewhere [2,6,104,105].

In vitro cell intoxication or infection with CDT, TT, or *pks*^+^ bacteria promotes apoptosis or senescence in a cell-dependent manner. T and B lymphocytes are very susceptible to CDT-induced apoptosis, while the majority of the other cell types tested undergo cell cycle arrest and eventually acquire a senescent phenotype [2]. Much less is known about the DDR outcomes in response to these effectors in vivo.

The experimental set ups for in vitro analysis of the BTGXs range from acute exposure from few to 72 h or “chronic” exposure >5 days, a time frame consistent with the acquisition of a senescent phenotype. It is important to note that the toxin doses used encompassed a very broad range (8 ng/mL up to 25 μg/mL). We do not know how these values compare to the “physiological” conditions, since we lack any information regarding the amount of toxin produced during a natural infection.

### 3.3. Pro-Inflammatory Effects of BTGX

Several lines of evidence indicate that intoxicated/infected cells produce a broad spectrum of pro-inflammatory cytokines. Secretion of IL-1β, IL-6, IFN-γ, and IL-8 in a dose-dependent manner (40 ng/mL–1 μg/mL) was induced by AactCDT as acute response (20 h) in peripheral blood mononuclear cells (PBMCs), with IL6 and IL8 being the most highly produced cytokines [106]. In line with these observations, IL1β, IL6, and TNFα were induced in TPA-differentiated human THP-1 cells and monocyte-derived macrophages exposed to low AactCDT dose (8–200 ng/mL) for 5 h, in absence of cell death induction [107]. Specifically, IL1β and IL18 secretion from the differentiated THP-1 cells was dependent on NLRP3 inflammasome and consequent caspase-1 activation [108].

The murine macrophage cell line RAW264.7 exposed to a CDT producing *A. actinomycetemcomitans* for 20h secreted IL-1β, IL12, and IL10 compared to cells exposed to an isogenic strain carrying a mutant toxin. The same secretory profile was induced in cells exposed to high AactCDT doses (6–25 μg/mL) [109]. Induction of pro-inflammatory mediators is not limited to cells of the immune system but has been also observed in cells of epithelial and mesenchymal origin. Indeed AactCDT induced secretion of IL-6 from resident fibroblasts [110], CjejCDT and infection with *H. hepaticus* expressing a functional genotoxin elicited IL8 secretion or mRNA production from the human embryo intestinal INT407 cells, as well as several colorectal-derived cell lines (T84, CaCo-2, HCA-7) 72 h post-treatment [111,112,113]. The latter was associated with NFκB activation and increased expression of inflammatory genes correlated with a Th17-dependent response (type 3 response) [113].

What are the molecular mechanisms linking BTGX-induced DNA damage and pro-inflammatory cytokines secretion? DNA damage is a danger to the cell and the host homeostasis. Recent evidence demonstrate how this danger is transduced in an inflammatory response, a conserved response to flag damage in most of the multicellular organisms from worms to humans [114]. It is well established that induction of DNA DSBs is a potent inducer of NFκB activation. This occurs via ATM-dependent phosphorylation of the NFκB essential modulator (NEMO), and consequent phosphorylation, ubiquitination and degradation of the NFκB inhibitor IκB [115] (Figure 4). In turn, this transcription factor plays a key role in induction of the pro-inflammatory IL1, TNFα, IL6, and IL8 [116]. Concomitantly CDT-induced DNA damage promotes also activation of the MAPK p38 [89] (Figure 5), which is also associated with induction of pro-inflammatory cytokine, such as IL1β, IL6, and TNFα [117].

Activation of transcription factors, within few hours from induction of DNA DSB, can account for the short term kinetic of pro-inflammatory cytokine secretion [118]. However, there is also a delayed effect [119], which is associated with two mechanisms: (i) detection of cytosolic self-DNA, an unusual location that is perceived as a threat (e.g., micronuclei, MN) [119]; (ii) acquisition of a senescence-associated inflammatory phenotype (SASP) [120] (Figure 4). The former triggers the cGAS-STING-mediated innate response leading to type I IFN production [119,121,122,123]. Indeed, cGAS (cyclic guanosine monophosphate (GMP)-adenosine monophosphate (AMP) synthase) is activated by sequence independent binding to cytosolic DNA, leading to production of the second messenger cyclic GMP-AMP (cGAMP). cGAMP is a high-affinity ligand for the endoplasmic reticulum-located STING (adaptor protein Stimulator of IFN Gene). Upon translocation to the Golgi apparatus, STING activates the TANK-binding kinase 1 (TBK1) and IFN regulatory factor 3 (IRF3) via a phosphorylation-dependent mechanism, and NFκB, resulting in transcription of type I IFN and other pro-inflammatory cytokines [124]. Cytosolic DNA fragments bound to MRE11 can also trigger the cGAS-STING pathway [125]. Since BTGXs promotes micronuclei formation in vitro [28,29,45,82,126], it is likely that these effectors can activate the cGAS-STING pathway, although this has not been experimentally addressed yet.

Cells that activate an efficient checkpoint response but fail to repair the DNA damage may undergo senescence [68,69,70,71] (Figure 5). This effect is well established in cells exposed to bacterial genotoxins in vitro [82,87,127,128], which have been shown to secrete a plethora of pro-inflammatory cytokines. Specifically, senescent human transformed and non-transformed cell lines secrete IL6, IL8 and IL24 in response to HducCDT [82]. Similarly, senescence induced by infection with *pks* positive *E. coli* promotes production of reactive oxygen species and secretion of pro-inflammatory cytokines, such as IL6, IL8, monocyte chemotactic protein (MCP)-1 and the matrix metalloproteinase (MMP)-3. The toxin-induced senescent phenotype is transmissible to bystander cells, indicating that the effect on the tissue microenvironment can be expanded beyond the single infected/intoxicated cells [87,127].

The inflammatory pattern observed in vitro has also been confirmed in in vivo experimental models. Presence of a functional CDT in *C. jejuni* enhances gastritis, gastric epithelial hypersplasia and mild dysplasia, and proximal duodenitis in mice deficient for the NFκB subunits (p50^−/−^ p65^+/−^, referred to as 3X mice) at 4 months post-infection (p.i.) compared to mice colonized with an isogenic *cdtB* mutant strain (in spite of a similar level of colonization). No difference was observed for hepatic lesions, pointing to a CDT tissue specific effect [30]. Interestingly, a Th2-mediated IgG1 serum response did not develop in C57BL/129 mice infected with the genotoxigenic *C. jejuni*, while both Th1 (IgG2a) and a Th2 (IgG1) responses were clearly observed in mice infected with a strain carrying a *cdtB* mutated gene [30].

Infection with genotoxigenic *H. hepaticus* or *H. cinaedi* promotes severe inflammation of the cecum and colon (typhlocolitis) in C57BL/6 IL10 deficient mice, a murine model for IBD [129,130,131]. The inflammation induced by the CDT producing *H. cinaedi* progressed to intestinal hyperplasia and dysplasia [130]. The typhocolitis was associated with a significantly higher induction of Th1 (IgG2c) and Th2 (IgG1) serum responses upon challenge with the wild type *H. hepaticus*, while animals challenged with the CDT-deficient mutant developed lower IgG2c responses and failed to mount the IgG1 response [129]. A similar pattern of serum IgG was observed in Swiss Webster mice exposed to the wild type *H. hepaticus* compared to that observed in mice infected with the *cdtB* mutant HhcdtBm7 strain [132]. The CDT-dependent pro-inflammatory effect was associated with persistent colonization of the intestinal tract with the CDT producing *H. hepaticus* in C57BL/6 interleukin 10 deficient (IL10^-/-^) (IL10-/-, at 8 months p.i.) and Swiss Webster mice (at 16 weeks p.i.) [129,132].

Interestingly, the presence of a functional HhepCDT was essential to promote dysplasia but did not enhance the severity of chronic hepatitis in A/JCr mice 10 months after infection [31]. However, the presence of a functional CDT promoted an enhanced inflammatory response at 4 months post-infection, prior the development of the dysplasia, characterized by enhanced transcription of NFκB subunits p50 and p65, and pro-the inflammatory mediators TNFα, IL-6, IFNγ, Cox-2, and anti-apoptotic Bcl-2 and Bcl-X(L) effectors in mice infected with the genotoxigenic *H. hepaticus* compared to mice infected with the control *cdtB* deficient strain [31]. A similar kinetic of transient upregulation (observed at 10 weeks p.i.) for *Il6* and *Tnfa* mRNA and reduced mRNA levels for the anti-inflammatory mediator *Il10* was associated with induction of intestinal neoplastic lesions (detected at 20 weeks p.i.) in 129/SvEv*Rag2^−/−^* mice infected with the wild type *H. hepaticus*. In the study, the presence of a functional CDT also correlated with an increased induction of DNA damage, assessed by detection of γH2AX [133]. These data suggest that the presence of a BTGX induces an inflammatory response, possibly due to a synergism between the CDT-induced DDR and the recognition of classical bacterial PAMPs (e.g., LPS) in the early stages of infection, which, in association with the ability of the toxin to promote genomic instability [28,29], fuels the carcinogenic process. We cannot exclude that the remodeling of the host immune response promotes an alteration of the intestinal microbiota and its transcriptomic profile, further contributing to a CDT-dependent alteration of the host intestinal microenvironment and immunological profile, as shown in *Apc^Min/+^* mice infected with *C. jejuni* wild type upon DSS treatment [32]. In these sets of experiments, mice exposed to the genotoxigenic *C. jejuni* presented enhanced tumor formation, similarly to the *H. hepaticus* study [133], and the microbial transcriptomic profiles and composition of the gut microbiota were significantly different compared to those observed in mice infected with an isogenic strain carrying a mutated *cdtB* gene [32].

In line with the alteration of tissue immune homeostasis and predisposition to an inflammatory phenotype, Secher and colleagues have shown that neonatal colonization of rats with *pks* positive *E. coli* impairs intestinal permeability, reduces the levels of resident T regulatory lymphocytes and tolerogenic DCs, increases levels of Th1-related IgG subclasses, and enhances mucosal delayed-type hypersensitivity response upon oral administration of the model antigen ovalbumin [47].

### 3.4. Immunosuppressive and Anti-Inflammatory Effects of BTGXs

In spite of the ample evidences linking the presence of a functional genotoxin with a pro-inflammatory environment, CDT, the first bacterial genotoxin identified, was initially described as an immunosuppressive factor [134]. This assessment was based on induction of apoptosis, inhibition of cell proliferation and IFN-γ secretion by activated human CD8^+^ and CD4^+^ T lymphocytes [134,135,136,137] (Figure 5).

Similar proapoptotic effects were induced on the T cell line Jurkat and primary splenocytes exposed to the *pks* positive *E. coli* [138]. This immunosuppressive activity was associated with an exacerbated lymphopenia and decreased survival rate in a model of septic infection with the *pks* positive pathogenic *E. coli* strain SP15 [138].

The immunosuppressive properties of CDT can also be exerted by affecting the viability of monocytes and immature monocyte-derived DCs, and impairing the stimulatory activity of activated DCs, the key activators of the adaptive immune responses [23,75,139,140,141] (Figure 5).

It is noteworthy that these immunosuppressive effects have been demonstrated in in vitro models, while limited data are available regarding the immune suppressive effects of BTGXs in the context of in vivo natural infections models in immunocompetent hosts.

Recent data showed that the expression of a functional typhoid toxin, derived from *S.* Typhi and expressed in *S.* Typhimurium, strongly suppresses the intestinal inflammation induced by the control *cdtB* deficient isogenic strain [142]. This effect has been confirmed in an infection model with the typhoid toxin-producing *S.* Javiana [143]. The anti-inflammatory outcome is tissue specific and observed only in the intestine, but not in liver and spleen, suggesting that the immunological profile of the microenvironment may affect the toxin immunomodulatory activity [142]. In line with this possibility, the presence of a functional toxin is associated with activation of a tissue-protective type 2 immune response, characterized by the presence of anti-inflammatory CD206^+^ macrophages, T regulatory lymphocytes, and enhanced expression of mRNA for Th2 cytokines (*Il10*, *Il4*, *Il13*, *Il5*) (Figure 5). The anti-inflammatory environment is induced in spite of a significant presence of senescent cells, uncoupling senescence from inflammation. This effect is dependent on a functional ATM, highlighting a direct interaction between the DDR and the regulation of the host response toward an anti-inflammatory effect (Martin et al., submitted).

Several lines of evidence support the anti-inflammatory role of the DDR, and specifically of ATM: (i) ATM deficiency increases the mortality rate upon DSS-induced colitis, concomitantly with upregulation of mRNA for proinflammatory cytokines (e.g., TNFα, IL12β, IL23, IL6) and higher percentage of activated CD44 positive T cells, a marker strongly associated with the Th17 population [144]; (ii) inhibition of ATM skews the polarization of T lymphocytes derived from rheumatoid arthritis patients toward Th1 and Th17, imposing a hyperinflammatory phenotype [145]; (iii) DNA damage induced by low doses of epirubicin induces an ATM-dependent protective effect in the lung tissue of septic mice, resulting in an enhanced survival [146].

Considering the tissue specificity of the anti-inflammatory effect of the typhoid toxin, it is conceivable that a tolerogenic immune microenvironment, like the intestine [147,148,149], imposes a different profile on the senescent secretome, due to activation of a specific set of transcription factors, determining a tissue-based education of senescence, which parallels the concept of tissue class control for the regulation of the immune response [100]. In support of this hypothesis, it has been shown that the JAK2-activated STAT3 transcription factor promotes an immune suppressive SASP in PTEN deficient prostate tumors [150]. The importance of the microenvironment in defining the effect of BTGXs on the host immune response is further corroborated by the demonstration that the typhoid toxin protective effect is lost when infection with the genotoxigenic *Salmonella* occurs in individuals with pre-existing colitis (Martin et al., submitted).

The immune-protective role of bacterial genotoxins has been also demonstrated for the *pks* island present in the probiotic *E. coli* strain Nissle 1917, where deletion of the *cblA* gene abolishes the genotoxin effect in vitro and the anti-inflammatory properties of the Nissle strain in two models of colitis [40]. It is noteworthy that deletion of *cblA* impairs not only the genotoxic activity but also the production of the microcins MccH47 and MccM, two antibacterial peptides associated with the probiotic activity of the Nissle 1917 strain. A single point mutation within the *clbP* gene (S95R) abolishes the DNA damaging effect without affecting the antibacterial activity in vitro and in an in vivo model of *Salmonella* Typhimurium infection [43]. Whether this activity is also sufficient for the *pks* anti-inflammatory effect in the colitis models needs to be further addressed.

## 4. Concluding Remarks

The interplay between the DDR and the IR may explain the puzzle regarding BTGXs, a family of effectors that cause DNA damage and senescence in most cell types in vitro and in vivo: a response that has a strong impact in the surrounding tissue microenvironment. These toxins have been shown to possess carcinogenic properties, however it is unlikely that bacteria have acquired these effectors to induce/promote cancer in the mammalian host.

It is possible that their primary role, at least at low toxins doses, is to modulate the host immune microenvironment in order to promote a suitable niche for the bacterium to replicate and spread. The final outcome (inflammatory vs anti-inflammatory response) may be dependent on the bacterium life-style and the tissue tropism. As corollary, the carcinogenic properties, demonstrated in vitro and in vivo, are collateral damage exerted under specific circumstances such as genetic susceptibility to cancer (haploinsufficiency of tumor suppressor genes, e.g., *APC*), inflammatory conditions (DSS-induced colitis or IL10 deficiency), and establishment of a chronic carrier status rather than an acute infection.

Many questions still need to be addressed, including the role of the immunological tissue profile in the definition of the SASP, the impact of chromatin alterations including γH2AX dynamics on SASP heterogeneity and the characterization of tissue and context-dependent types of senescence. These issues can be addressed by performing a detailed multiplex transcriptomic analysis in situ, which allows simultaneous characterization of cell-specific and senescence markers, transcription factors, and cytokines, maintaining the tissue architecture and the spatial relation between cells.

Another question concerns the levels and the regulation of BTGX production in vivo and how these levels are comparable with the doses used in the in vitro experiments. Payros and colleagues have recently demonstrated that food contaminated with the mycotoxin deoxynivalenol (DON) exacerbates the genotoxic effect induced by *pks* positive *E. coli* in vitro and upon colonization in an in vivo model [151]. These data indicate that environmental factors can affect the outcomes or the severity of BTGX exposure. Although the authors have not assessed the DON effect on the levels of expression of the *pks* genes, this is a possibility that needs to be considered.

In spite of the fact that more than 30 years have passed since the first characterization of CDT activity in *E. coli* and *Shigella* ssp [152,153], we still have a long, complex but very fascinating journey ahead.
